# Supplementary material for: Iso-orientation bias of layer 2/3 connections unifies spontaneous, visually and optogenetically driven V1 dynamics
Source: Nat Commun. 2026 Jan 21;17:1901. doi: 10.1038/s41467-026-68578-y (PMC12923880; doi:10.1038/s41467-026-68578-y)
Supplement: Supplementary file 2 — Description of Additional Supplementary Files [file 41467_2026_68578_MOESM2_ESM.pdf]

Description of Additional Supplementary Files

Supplementary Movie 1: Example video of the generated LFP signal of the model Layer 2/3 spontaneous activity showing propagation of a Spontaneous Travelling Wave
